# In situ decoration of graphene sheets with gold nanoparticles synthetized by pulsed laser ablation in liquids

**DOI:** 10.1038/srep30478

**Published:** 2016-07-28

**Authors:** Rafael Torres-Mendieta, David Ventura-Espinosa, Sara Sabater, Jesus Lancis, Gladys Mínguez-Vega, Jose A. Mata

**Affiliations:** 1 GROC·UJI, Institute of New Imaging Technologies, Universitat Jaume I, Avda. Sos Baynat s/n, 12071, Castellón, Spain; 2 INAM, Institute of Advanced Materials, Universitat Jaume I, Avda. Sos Baynat s/n, 12071, Castellón, Spain

## Abstract

The demand for nanocomposites of graphene and carbonaceous materials decorated with metallic nanoparticles is increasing on account of their applications in science and technology. Traditionally, the production of graphene-metal assemblies is achieved by the non-environmentally friendly reduction of metallic salts in carbonaceous suspensions. However, precursor residues during nanoparticle growth may reduce their surface activity and promote cross-chemical undesired effects. In this work we present a laser-based alternative to synthesize ligand-free gold nanoparticles that are anchored onto the graphene surface in a single reaction step. Laser radiation is used to generate highly pure nanoparticles from a gold disk surrounded by a graphene oxide suspension. The produced gold nanoparticles are directly immobilized onto the graphene surface. Moreover, the presence of graphene oxide influences the size of the nanoparticles and its interaction with the laser, causes only a slight reduction of the material. This work constitutes a green alternative synthesis of graphene-metal assemblies and a practical methodology that may inspire future developments.

Graphene sheets decorated with metal nanoparticles (MNPs) are excellent materials that can be used in several fields such as energy storage, optoelectronics, biosensors or catalysis, due to their physical and chemical properties^1^,^2^. The combination of the unique graphene conductivity properties and the high reactivity of MNPs^3^,^4^,^5^, results in the ability to control phenomena such as the spatial localization of the surface plasmon resonance of the metallic particles^6^, the enhancement of light absorption of graphene sheets^7^, the bio-conjugation with a variety of molecules to produce biosensors^8^,^9^,^10^,^11^,^12^, and the improvement of the catalytic activity of the nanoparticles^13^,^14^.

The vast majority of graphene-MNPs are synthesized by the simultaneous reduction of metal salts and highly oxidized graphene derivatives^15^,^16^,^17^. This methodology involves the use of co-ligands or surfactants on the formed nanoparticles, in order to enhance their stability and avoid aggregation^18^,^19^. This synthetic route produces chemical waste along the whole process and in the final product^20^,^21^: the MNPs are usually covered with surface adsorbed ligands that promote cross-chemical effects^14^.

Ligand-free MNPs have been successfully prepared by laser irradiation using an alternative and easy top-down technique that fulfils the principles of green chemistry^22^,^23^. Pulsed Laser Ablation in Liquids (PLAL) is based on the irradiation of a focused pulsed laser beam onto the surface of a solid target surrounded by almost any kind of liquid or colloid^24^,^25^,^26^,^27^. The interaction between the strong electromagnetic field of the laser radiation and the atoms at the material’s surface, promotes the extraction of electrons in the surface creating an electronic cloud and leaving a deficiency of electrons in a localised area in the target’s surface. The ions in the surface are attracted by the electronic cloud through electromagnetic forces, resulting in the detachment of bulk material in the form of atom clusters, large fragments or simply ionized atoms. After this process, a cavitation bubble is formed confining the material that forms crystalline NPs and eventually, when the cavitation bubble is extinguished, the NPs are dispersed into the liquid ambiance^28^,^29^. Considering that ligand-free gold NPs present an improved specific surface activity due to the absence of ligands on the surface, the particles can be retained in the surface of graphene sheets by the combination of different effects: (i) the electrostatic interactions between the NPs and graphene lead to a strong immobilization, (ii) the π-electron aromatic system found in graphene and its derivatives interacts with the d-orbitals of the MNPs^30^ or (iii) the selective distribution of nanoparticles at the defects found in graphene^31^.

Decoration of ligand-free gold NPs created separately by PLAL and subsequently mixed with graphene derivatives has been reported^32^. Nevertheless, the possibility to produce particles directly in a liquid medium filled with graphene oxide sheets implies that the electromagnetic field of the laser beam promotes a reactive process that may influence the reduction degree of graphene oxide^33^,^34^. In this way, the laser radiation removes oxygen functional groups from the graphene oxide sheets, while keeping the characteristic wide interlayer spacing that characterizes the electronic decoupling of individual layers in this material, which in some cases leads to superconductivity effects^35^,^36^.

In this work, we have focused on producing ligand-free gold NPs anchored to graphene oxide sheets in a single reaction step. The impact of the femtosecond radiation-based technique on the control of the production of ligand-free gold NPs in an environment filled with graphene oxide sheets has been studied. With this aim, AuNPs were produced in two different liquid environments for comparison purposes: (i) deionized water and (ii) a graphene-oxide homogeneous suspension. Our results demonstrate that it is possible to create ligand-free MNPs directly anchored to graphene oxide sheets in a single reaction process. This methodology has been proved to provide an effective way to obtain graphene metal assemblies that could potentially be used in several applications where undesirable cross chemical effects need to be avoided.

## Results

### Laser synthesis of AuNPs using water as solvent

**Figure 1:**
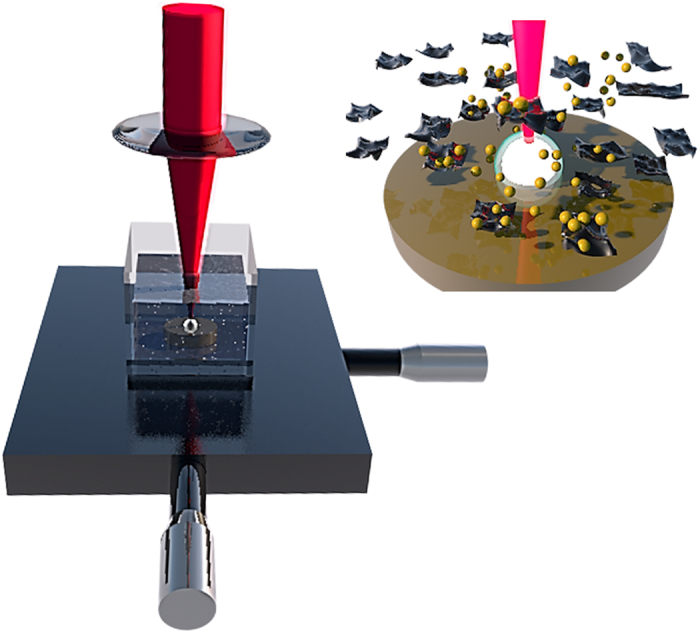
Experimental set-up for the synthesis of NPs.

Gold nanoparticles were obtained from the ablation of a gold solid target immersed in deionized water using an amplified Ti:Sapphire femtosecond laser source working at a maximum energy per pulse of 0.8 mJ, with a pulse duration of 30 fs full width half maximum and 1 kHz of repetition rate. The schematic drawing of the set-up is presented in Fig. 1 (for a detailed description of the experiment preparation see the Methods section and Supplementary Movie 1). The synthesis of AuNPs is usually influenced by physical and chemical processes. The physical processes are generally governed by the laser radiation power, the pulse duration, the repetition rate and the way in which the beam is driven to the target; and the chemical processes are governed by the interaction between the radiation and the base fluid.

**Figure 2:**
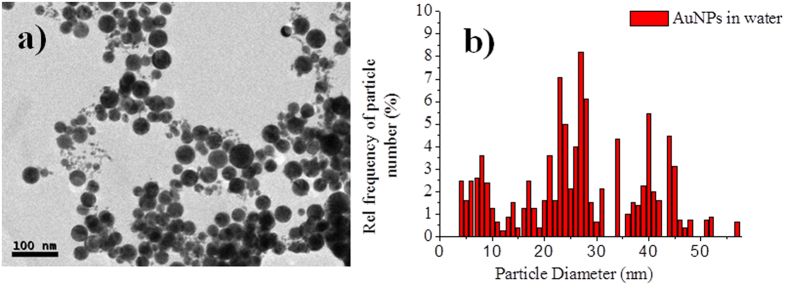
AuNPs produced in H_2_O: (**a**) TEM micrograph and (**b**) histogram of the size distribution of the NPs. Statistical analysis done with 324 particles.

The micrograph displayed in Fig. 2 shows the spherical morphology of the particles synthesized by laser ablation in water at a fluence of ~1 J/cm^2^. This is the characteristic MNP shape obtained through the PLAL process. During laser radiation, fragmented material is released from the bulk material and suspended in the liquid medium with a certain density. The average size of the obtained gold particles was 26 nm with a size distribution of 18 nm (Fig. 2b).

**Figure 3:**
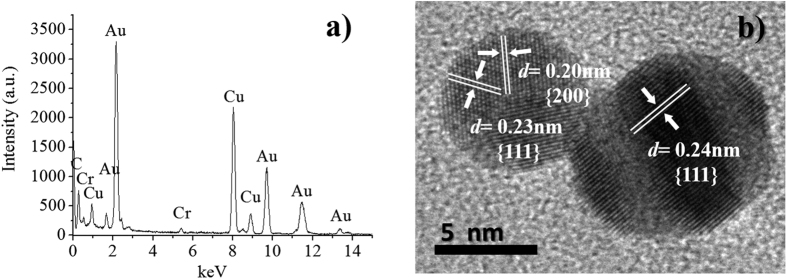
(**a**) EDX spectrum of the produced AuNPs, (**b**) HRTEM micrograph of AuNPs showing the inter-planar spacing, *d*.

The EDX spectrum shown in Fig. 3a reveals the presence of elements like copper (Cu), carbon (C), chromium (Cr), iron (Fe) and gold (Au). The presence of copper and carbon is caused by the TEM grid, and Fe and Cr are detected as components of the microscope lenses. As it can be extrapolated from these results, the synthesized material seems to be free from any other element or chemical impurity. This observation is further supported by the HRTEM micrographs depicted in Fig. 3b, where the lattice fringes indicates the high crystallinity of the material, which allows determining the inter-planar spacing, *d*, represented by the {*hkl*} Miller index. The *d*-spacing of 0.02 nm corresponds to the {200} lattice plane of gold, and the 0.23 nm and 0.24 nm spacings to the {111} lattice plane of gold. The obtained results are similar to those reported in the Joint Committee on Powder Diffraction Standards (JCPDS) files for cubic Au crystals (JCPDS: 4-784).

### Laser synthesis of AuNPs immobilized on GO

The decoration of graphene oxide sheets with AuNPs was achieved through the PLAL process by radiation a gold disk in a graphene oxide (GO) suspension in deionized water (1 mg/mL). The experimental set-up and conditions were exactly the same as those previously described using water as the medium. As such, the fluence was set to ca. 1 J/cm^2^. The immobilization of ligand-free AuNPs happens in a natural way after particle formation. The particles are formed in an environment filled with the carbonaceous material, which becomes a support for the newly formed nanoparticles.

**Figure 4:**
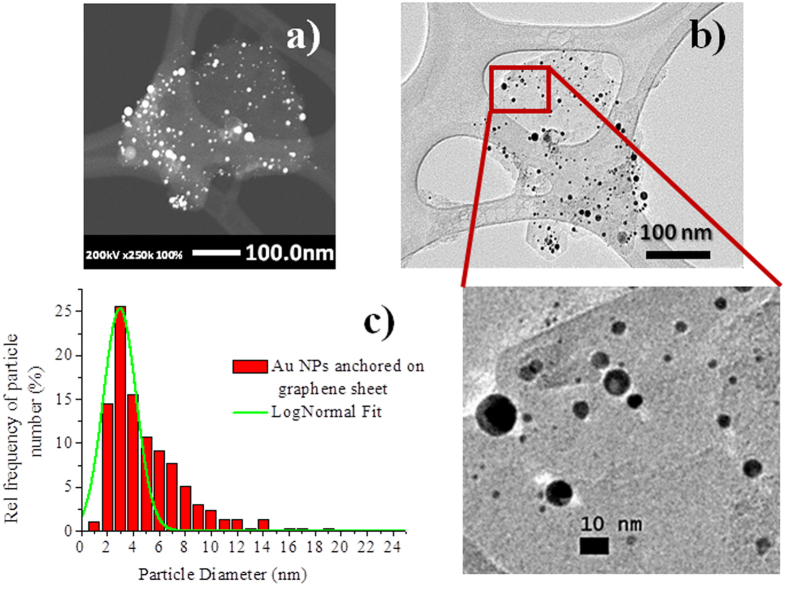
AuNPs anchored on graphene sheets, (**a**) DF-STEM micrograph, (**b**) TEM micrograph and (**c**) histogram of the NP size distribution. Statistical analysis done with 650 particles.

The size of the particles, the morphology and the purity of the AuNP/GO material were characterized by TEM, EDX, Fast Mapping with SDDs (FS Mapping) and Dark-Field Scanning Transmission Electronic Microscopy (DF-STEM). The micrographs depicted in Fig. 4 show the spherical morphology of the particles synthesized by the laser procedure. The shape of the NPs was also confirmed by DF-STEM analysis with an HAADF (high-angle annular dark field) detector for an improved resolution. The characteristic shape obtained is most probably due to the PLAL process and the phenomena that lead to the spherical shape of the particles, in the same manner as for the NPs obtained in the experiment using just water.

A statistical analysis showed that the average size of the AuNPs in the final material is 3 ± 1.1 nm. The majority of the particles are in the average size, but a few particles of size >20 nm were also found in the sample. The average size distribution achieved is lower than that using only water as the solvent. When water was used as the medium, the average particle size was 26 (Fig. 2b). Under the same conditions, GO has an important influence on the size of the AuNPs.

**Figure 5:**
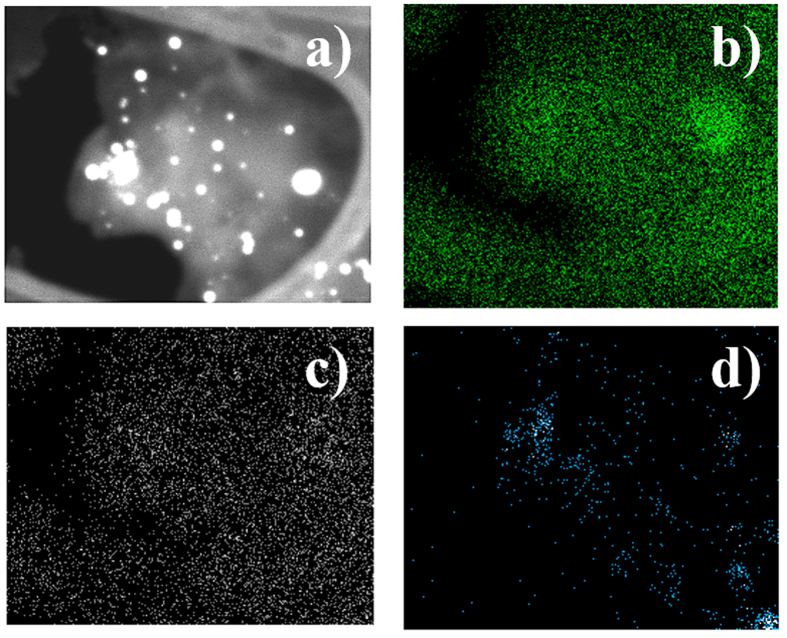
FS Mapping of AuNPs anchored on graphene sheets: (**a**) DF-STEM micrograph where the mapping was taken, (**b**) emission of carbon, (**c**) emission of oxygen, (**d**) emission of gold.

The FS Mapping measurements, obtained by means of the EDX system and the special HAADF detector, show that this laser-assisted fabrication route produces highly pure AuNPs anchored on GO with an heterogeneous distribution (Fig. 5). The DF-STEM emission of oxygen and carbon (Fig. 5b,c) represents a homogeneous distribution but gold (Fig. 5d) is located in certain regions. These results suggest that there is a major concentration of AuNPs located at the graphene sheets with a high density of wrinkles (Fig. S3).

**Figure 6:**
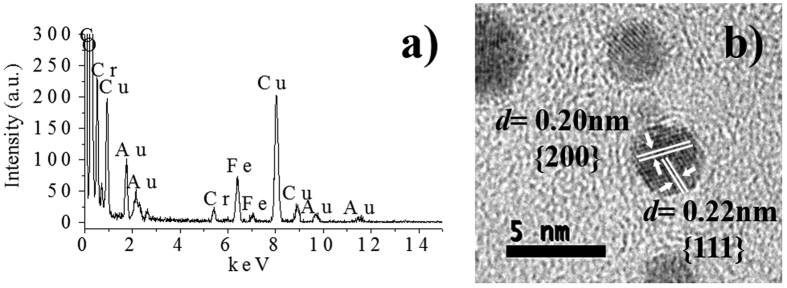
(**a**) EDX spectrum of the AuNP/GO hybrid material. (**b**) HRTEM micrograph of AuNPs immobilized in GO sheets showing the inter-planar spacing, *d*.

The EDX spectrum shows that, when the irradiated area by the electron beam is large, some extra elements (such as Cu, C, Cr and Fe) can be detected, similarly to what was observed in the samples produced in water (Fig. 6). The most evident difference is the intensity peak corresponding to carbon. This increase is attributed to the presence of graphene sheets. The HRTEM analysis (Fig. 6) shows that the synthesized AuNPs/GO material is free of any other element or chemical impurity. This observation is further supported by the HRTEM (Fig. 6b), where the lattice fringes indicates the high crystallinity of the material, which allows determining the inter-planar spacing, d. The Miller index found correspond to those reported in the Joint Committee on Powder Diffraction Standards (JCPDS) files for cubic Au crystals (JCPDS: 4-784).

**Figure 7:**
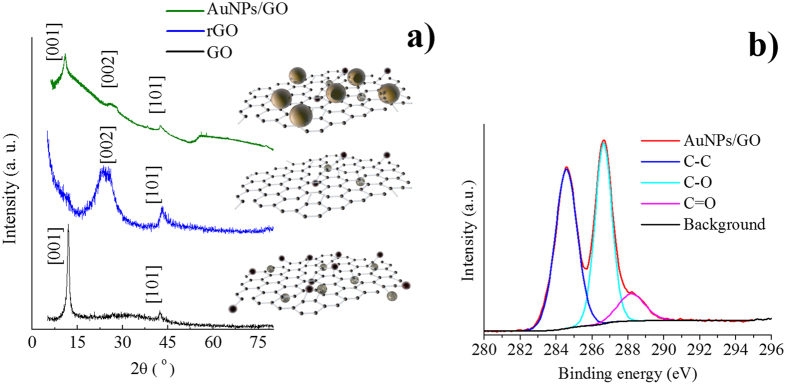
(**a**) XRD patterns of AuNP/GO, rGO and GO, (**b**) C1s XPS spectra of AuNPs/GO.

The interaction between a high power electromagnetic field, such as the one associated to femtosecond laser radiation, and almost any kind of material promotes a considerable interchange of energy that can result in structural changes in the material. In order to study how femtosecond laser radiation centered at 800 nm modifies the graphene oxide sheets, the AuNP/GO material was further analyzed by powder X-ray diffraction. Figure 7 displays the XRD patterns of GO, reduced GO (rGO, obtained using hydrazine)^37^ and AuNP/GO. The GO pattern shows two peaks, an intense peak at 2θ = 12° and a low intensity peak relative to the first one at 42.65°. The first peak is associated with the crystalline plane [001], which arises from an increase in the interlayer distance compared to that of graphite as a consequence of the oxygen functionalities introduced in the carbonaceous material after oxidation^38^ The second peak corresponds to the crystalline plane [101], also present in graphite (the Miller index and *d* inter-planar spacing for hexagonal graphite are included in the Joint Committee on Powder Diffraction Standards (JCPDS): 41-1487).

The XRD pattern of rGO is completely different, the [001] signal disappears; however, the peak corresponding to the [002] plane appears at 24.85°, and the peak for [101] is observed at 43.1°. The reduction of GO using hydrazine to obtain rGO removes many oxygen functionalities and restores part of the π-network of the material^39^. The XRD pattern for AuNP/GO shows an intermediate situation between GO and rGO. A peak at 2θ = 10.85°, corresponding to the [001] crystalline plane, is observed, and so are the peaks corresponding to the [002] and [101] planes. These results suggest that laser irradiation has an effect on the graphene material. The reduction degree of the AuNP/GO material was further analyzed by XPS^40^. The presence of C-C (286.4 eV), C-O (286.6 eV), and C=O (288.2 eV) functional groups allows us to determine a C/O ratio of 2.4 for the AuNP/GO. In the parent graphene oxide the C/O ratio is 2.0 and in the case of highly reduced graphene oxide (rGO), prepared by reduction of GO using hydrazine, the C/O ratio is 10.4. These results are in agreement with the XRD analysis and suggest that a slight reduction of graphene oxide is produced after laser irradiation. Details of XPS analysis corresponding to GO and rGO are in the Supplementary Material.

## Discussion

Using PLAL, we have decorated graphene oxide sheets with ligand-free AuNPs in a practical and single step reaction. The laser-based method causes a slight reduction of graphene oxide but avoids the use of extra chemicals that can alter the properties of the gold nanoparticles. The irradiation of a gold disk containing a GO suspension in water leads to the immediate immobilization of the generated ligand-free AuNPs on the graphene surface. According to the experimental set-up (Fig. 1), the laser radiation interacts with the suspension of graphene oxide before reaching the gold disk. In order to establish any effect from the laser radiation on the graphene material, the AuNPs were prepared in two different liquid environments, *i.e.* deionized water and a graphene oxide suspension in water.

Comparison of the results of both experiments suggests that graphene oxide has an influence on the AuNP formation and in graphene oxide itself by i) decreasing the average particle size, ii) immobilizing the particles on the graphene surface, iii) heterogeneously distributing the anchored AuNPs at the graphene wrinkles, and iv) slightly reducing the graphene oxide.

The presence of graphene oxide has an influence on the size of the gold nanoparticles. The decrease of particle size can be explained by considering graphene oxide as an optical density filter and as a capping agent. GO acts as a neutral density filter avoiding the delivery of the full input irradiation fluence ~1 J/cm^2^ onto the surface of the gold target disk. The effect of graphene oxide as a density filter was evaluated experimentally by performing an additional experiment: AuNPs were produced using only water as the medium at a fluence of ~0.25 J/cm^2^ which is the fluence calculated to reach the gold disk through the GO dispersion (Section 1, Supplementary Material). The particles obtained using this procedure have an average size of 6 nm. This value is significantly different from the average value obtained using graphene oxide of 3 nm for considering that the graphene oxide is acting only as a neutral density filter. New experiments were necessary to further evaluate the effect of graphene oxide concentration on the size of the Au NPs. The experiments were carried out using the standard conditions described in the laser synthesis of AuNPs and variable concentrations of GO in deionized water (Table S1). The results show that for all concentrations of graphene oxide the size of the gold nanoparticles is in the range of 2–5 nm. Apparently, there is not a significant effect on the size of Au NPs when varying the concentration of graphene oxide. These results suggest that graphene oxide is also acting as a capping agent avoiding the growth of the nanoparticles.

Another interpretation for the decrease of the particle size was to consider the selective immobilization of AuNPs on the surface of graphene. Laser irradiation produces particles of different sizes, but it could be expected that only the small particles are retained on the surface of graphene, leaving the rest suspended in the liquid. In order to confirm that “big” AuNPs can also be anchored on the surface of graphene, a complementary experiment was carried out. Using the protocol in water and maintaining the fluence at ~1 J/cm^2^, we obtained particles with an average size centered at 26 nm and a size distribution of 18 nm. Graphene oxide (1 mg/mL) was then added to the resulting solution and the mixture was stirred for 12 h. TEM analysis revealed that all the AuNPs previously formed in water had been immobilized onto the graphene oxide surface (results shown in the Supplementary Material). Most interesting is the observation that even the ‘big’ AuNPs are immobilized on the surface of the material. These experiments suggest that, when a gold disk is irradiated in the presence of GO smaller AuNPs are obtained. Most likely, GO is partially blocking the pathway of the laser beam, acting as a neutral optical density filter and as a capping agent avoiding nucleation. Graphene oxide promotes the immobilization and controls the size of the AuNPs formed by the PLAL process.

In the TEM analysis of the samples obtained using GO, we have observed a heterogeneous distribution of AuNPs. The micrographs in Fig. 4 show the characteristic single-to-few layers nature of the graphene oxide sheets with some wrinkles. We noticed that the presence of wrinkles facilitates the adsorption of the AuNPs. This observation is based on the heterogeneous distribution of AuNPs: we found a larger concentration of AuNPs close to, or at, the wrinkles than in other areas of the material (Section 4, Supplementary Material). The wrinkles found in GO correspond to areas with a high concentration of defects, which is the most probable cause for the high accumulation of NPs in these areas. The forces contributing to the AuNPs immobilization on the surface of the material are a combination of electrostatic interactions, overlapping of the extended π-orbitals of graphene oxide with the d-orbitals of the metal nanoparticles, and/or the selective distribution of particles at the defects of the graphene oxide sheets^41^,^42^,^43^.

The powder XRD and XPS revealed that graphene oxide is slightly reduced through the interaction with the laser radiation. This effect is observed in the XRD pattern of AuNP/GO, which displays the peak corresponding to the crystalline plane [001] (indicative of the presence of oxygen functionalities in GO), but also the peak corresponding to the [002] plane (denoting a partial restoration of the π-network). These results are further confirmed by XPS. After laser radiation, we have observed an increase of C/O ratio indicating that there is a decrease of oxygen functional groups. In this work, we have shown the convenience of using a laser-based method for the production of ligand-free AuNPs that are directly immobilized on the surface of graphene oxide. The modulation of laser properties and the presence of the graphene material have an influence in the size of the AuNPs. The properties of graphene oxide are not altered considerably after laser irradiation and we have only observed a slight increase in the reduction degree of the material. Considering previously reported results, we can make a comparison of gold nanoparticles anchored onto graphene obtained by chemical methods and laser ablation. We have observed that chemical methods allow a better control of the size dispersion of nanoparticles and produce a complete reduction of GO^44^,^45^ When using laser ablation for the synthesis of gold nanoparticles, the GO is only slightly reduced and the inherent properties are preserved. The graphene acts as a capping agent and reduces the size of the nanoparticles in comparison with the formation of nanoparticles using water. We believe that the simplicity of this procedure for AuNPs/GO synthesis may inspire future developments in the field of materials science.

## Methods

### Laser synthesis of AuNPs in both water and GO media

In order to produce the nanomaterials and understand the influence of graphene sheets in the synthesis of AuNPs, two different experiments were performed. For the first experiment, deionized water (18 MΩ) was used as the liquid medium to fabricate gold NPs by the *in-situ* femtosecond laser radiation strategy known as PLAL. The particles obtained using this procedure are in agreement with previous reported literature^46^. For the second experiment, a mixture of deionized water (18 MΩ) and graphene oxide (GO) was used as the liquid medium to fabricate the AuNPs and decorate the graphene sheets in a single step. GO was prepared from graphite powder (natural, universal grade, 200 mesh, 99.9995%) by the Hummers method^47^.

In all cases, a gold disk of thickness 1 mm and diameter 6.5 mm (99.99% purity) was used as a target to produce ablation. The target was surrounded by the liquid medium (H_2_O and H_2_O + GO respectively) and the ejected material that comes from the ablation process was captured in the surrounding medium. The gold disk was placed at the bottom of a glass chamber filled with H_2_O for the first experiment and H_2_O + GO for the second. The thickness of the fluid layer above the gold target in all cases was set to be ~2 mm. Then the target was irradiated from the air-liquid interface as shown in Fig. 1 with the femtosecond laser beam focused with a 35 mm lens onto the target surface plane while it is moving at a scanning constant velocity of 0.45 mm/s by means of a 2D motion controlled stage to ensure same focusing conditions over the target’s surface.

The experiments were carried out with a Ti:Sapphire laser (Femtopower Compact Pro, Femtolasers) emitting pulses of 30 fs, with a central wavelength of 800 nm, a maximum energy per pulse of 0.8 mJ, and 1 kHz repetition rate. The delivered energy was monitored by means of an analogical power meter (Spectra Physics, Model 407-A) and controlled using a set of calibrated neutral density filters. The diameter of the incoming laser beam was controlled using an iris of 6 mm of diameter placed before the focusing lens.

The optimized fluence of the laser beam at the sample plane was set to ~1 J/cm^2^ in order to avoid the plasma breakdown of water, while having enough energy to promote ablation. For the complimentary experiment where GO was considered a neutral optical density filter, we replaced the absorption contribution of H_2_O + GO by a neutral optical density filter which promotes the same power decrement, achieving this way a fluence of ~0.25 J/cm^2^ in the target. The dispersion introduced by either of the liquids, and the optical elements in general, was partially compensated in a post-compression stage by changing the relative position of two fused silica Brewster prisms, which guaranteed the minimum pulse duration at the sample.

### Sample preparation for analysis

The samples were analyzed by X-ray diffraction and electronic microscopy. High Resolution Transmission Electron Microscopy (HRTEM) images and High-Angle Annular Dark-Field (HAADF)-STEM images of the samples were obtained using a Jem-2100 LaB6 (JEOL) transmission electron microscope coupled with an INCA Energy TEM 200 (Oxford) energy dispersive X-ray spectrometer (EDX) operating at 200 kV. The preparation of the samples for TEM measurements was carried out by deposition of a droplet of the colloidal suspension onto a carbon-coated copper-based TEM grid. The liquid content was removed with the help of absorbent paper and, then, the sample was air-dried for several hours. The solid material collected on the grid surface was used for analysis. This technique allows the preparation of dry and highly dispersed particles. The crystallographic structure of the samples was analyzed by X-ray powder diffraction (XRD) using a Bruker D4-Endeavor diffractometer with Cu-Kα radiation at a wavelength of 0.1542 nm. X-ray photoelectron spectroscopy (XPS) spectra were acquired on a Kratos AXIS ultra DLD spectrometer with a monochromatic Al Kα X-ray source (1486.6 eV) using a pass energy of 20 eV. The photoelectron take off angle was 90° with respect to the sample plane. To provide a precise energy calibration, the XPS binding energies were referenced to the C1s peak at 284.6 eV.

## Additional Information

**How to cite this article**: Torres-Mendieta, R. *et al*. In situ decoration of graphene sheets with gold nanoparticles synthetized by pulsed laser ablation in liquids. *Sci. Rep.* **6**, 30478; doi: 10.1038/srep30478 (2016).

## Supplementary Material

Supplementary Information

Supplementary Video S1
