# Supplementary material for: In situ decoration of graphene sheets with gold nanoparticles synthetized by pulsed laser ablation in liquids
Source: Sci Rep. 2016 Jul 28;6:30478. doi: 10.1038/srep30478 (PMC4964631; doi:10.1038/srep30478)
Supplement: Supplementary Information [file srep30478-s1.pdf]

Supplementary Information for:

In situ decoration of graphene sheets with gold nanoparticles synthetized by pulsed laser ablation in liquids

Rafael Torres-Mendieta,[a] David Ventura-Espinosa,[b] Sara Sabater,[b] Jesus Lancis,[a] Gladys Mínguez-Vega[a,*] and Jose A. Mata[b,*]

[a]GROC·UJI, Institute of New Imaging Technologies, Universitat Jaume I, 12071-Castellón (Spain).

[b]INAM, Institute of Advanced Materials, Universitat Jaume I, Avda. Sos Baynat s/n, 12071, Castellón (Spain).

1. Particles created in GO+H_2O using a fluence of ~0.25 J/cm^2.
2. Size of AuNPs vs. graphene concentration
3. Particles created in H_2O using a fluence of ~1 J/cm^2 and subsequent immobilized in GO.
4. Observation of heterogeneous particle distribution along graphene sheets.
5. Supplementary XPS spectra.
6. HRTEM of silver nanoparticles on GO obtained by laser ablation.
7. Supplementary references.
8. Video: Experimental set up for the synthesis of gold nanoparticles and immobilization onto graphene

1. Particles created in GO + H_2O using a fluence of ~0.25 J/cm^2.

Considering that graphene oxide was acting as a neutral optical density filter in the synthesis process, we have produced Au-NPs using a diminished energy but keeping the standard conditions. The optical density was calculated by measuring the radiation absorption of the laser beam with an analogical power-meter (Spectra Physics, Model 407-A) in a rectangular quartz cuvette. The 2 mm layer of H_2O + GO above the gold disk was filtering the 75 % of the input radiation, consequently, the fluence delivered to the target is ~0.25 J/cm^2.

In Figure S1, we show the TEM micrograph corresponding to the AuNPs obtained in water using a fluence value of ~0.25 J/cm^2. The histogram shows that changing the fluence value modifies the particle size and the size distribution. Using a fluence value of ~0.25 J/cm^2 the average size of the gold nanoparticles is 6 nm.

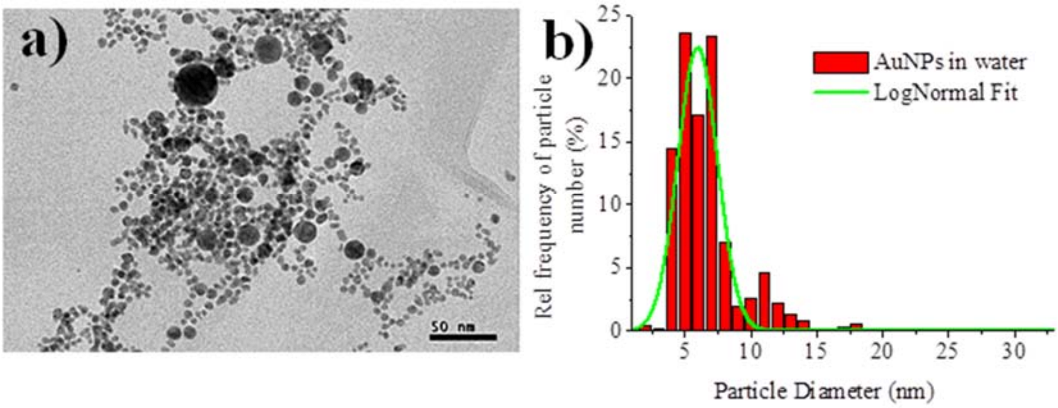

Figure S1. Micrograph of Au-NPs produced in H_2O using a fluence of ~0.25 J/cm^2, a) TEM micrograph and b) histogram of the size distribution of NPs. Statistical analysis done with 434 particles.

These results are in agreement with previous publications. Different studies demonstrate that using energies below 65 μJ the normal average size for the produced particles is in the range of 7 - 10 nm [1].

2. Size of AuNPs vs. graphene concentration

Table S1: Effect of graphene oxide concentration on the size of gold nanoparticles.

Concentration **0.1 mg/mL**

HRTEM image	Histogram	Number Particles	Size (nm)
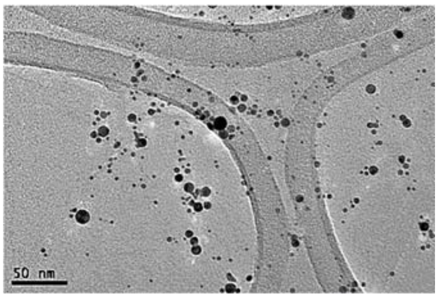	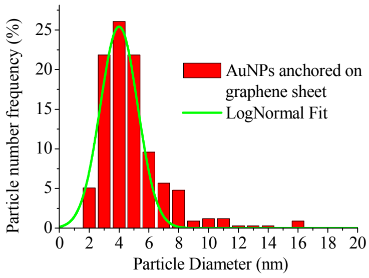	334	4.0 ± 1.5

Concentration **0.25 mg/mL**

HRTEM image	Histogram	Number Particles	Size (nm)
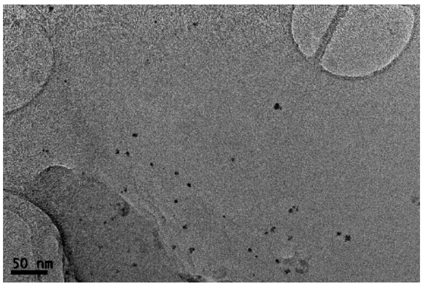	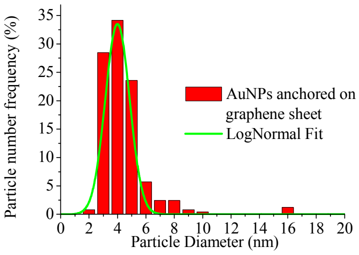	246	4.0 ± 1.0

Concentration **0.5 mg/mL**

HRTEM image	Histogram	Number Particles	Size (nm)
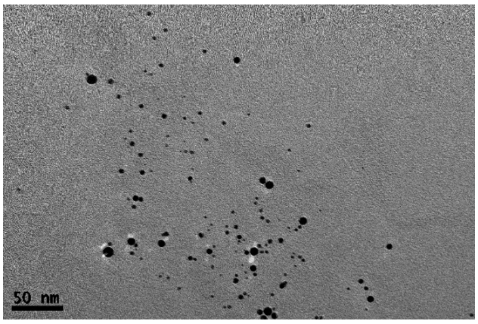	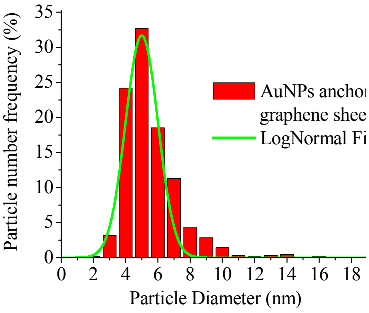	637	5.0 ± 1.2

Concentration **1 mg/mL**

HRTEM image	Histogram	Number Particles	Size (nm)
		650	3.0 ± 1.1

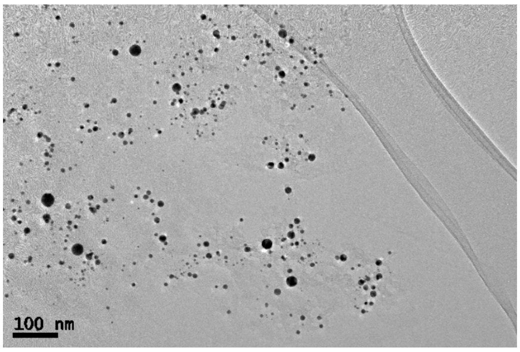

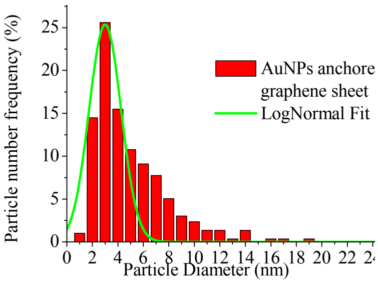

Concentration **2 mg/mL**

HRTEM image	Histogram	Number Particles	Size (nm)
		91	3.0 ± 1.3

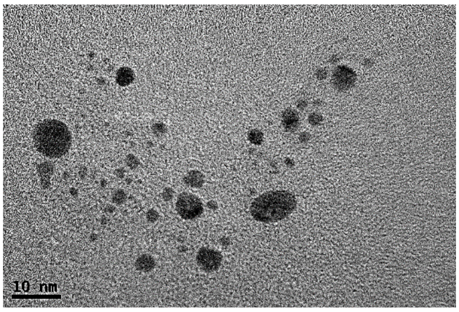

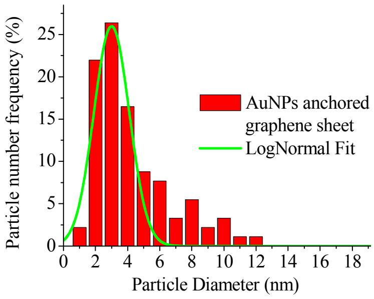

We observed that there is not a significant change in the size of the AuNPs when modifying the graphene concentration. When the AuNPs are created, they are immediately anchored to the GO sheets that alters their coalescence process. Consequently, we think that the graphene oxide sheets are acting as a capping agent and change the normal mechanism of NPs growth.

3. Particles created in H_2O using a fluence of ~1 J/cm^2 and subsequent immobilized in GO.

This experiment serves to demonstrate that the small average size particles anchored graphene sheets was not a consequence of selective immobilization. To do this, Au-NPs were created in water using a fluence of ~1 J/cm^2 and keeping the standard conditions. The particles were further mixed with Graphene oxide (1 mg/mL) and the mixture was stirred for 12 h. The results depicted in Figure S2 show that particles with sizes above 34 nm were also anchored to the graphene surface, demonstrating in this way that bigger particles can be anchored to graphene sheets and pointing out that the influence of graphene oxide in the Au-NPs synthesis is the promotion of lowering the average particle size.

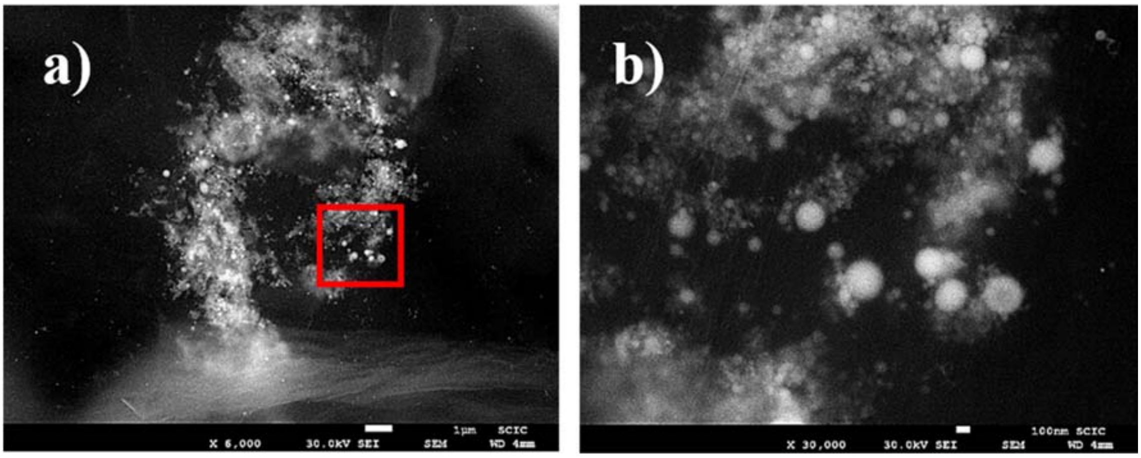

Figure S2. Micrograph of Au-NPs hooked in graphene sheets, a) SEM micrograph and b) zoom of the red square in figure a) showing the size of particles clearly bigger than 34 nm.

4. Observation of heterogeneous particle distribution along graphene sheets.

From TEM micrograph statistical analysis, it was determined that the highest concentration of Au-NPs was found where the highest wrinkle concentration was present in the graphene surface (Figure S3). The wrinkles presence is due to crystalline defects in the graphene sheets and the high concentration of NPs in or close to this area can be explained as a combination of electrostatic interactions, overlapping of the extended π-orbital of graphene oxide with the d orbitals of the metal NPs and/or allocation of particles at the defects of graphene oxide sheet. [2]

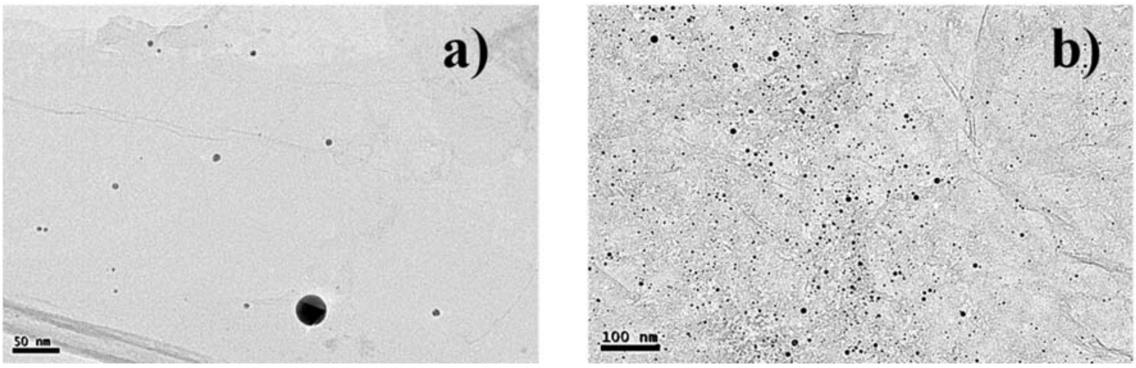

Figure S3. Micrographs of Au-NPs hooked in graphene sheets, a) graphene sheet with a low density of wrinkles and b) graphene sheet with a high density of wrinkles.

5. Supplementary XPS spectra

X-ray photoelectron spectroscopy (XPS) spectra were acquired on a Kratos AXIS ultra DLD spectrometer with a monochromatic Al Kα X-ray source (1486.6 eV) using a pass energy of 20 eV. The photoelectron take off angle was 90º with respect to the sample plane. To provide a precise energy calibration, the XPS binding energies were referenced to the C1s peak at 284.6 eV.

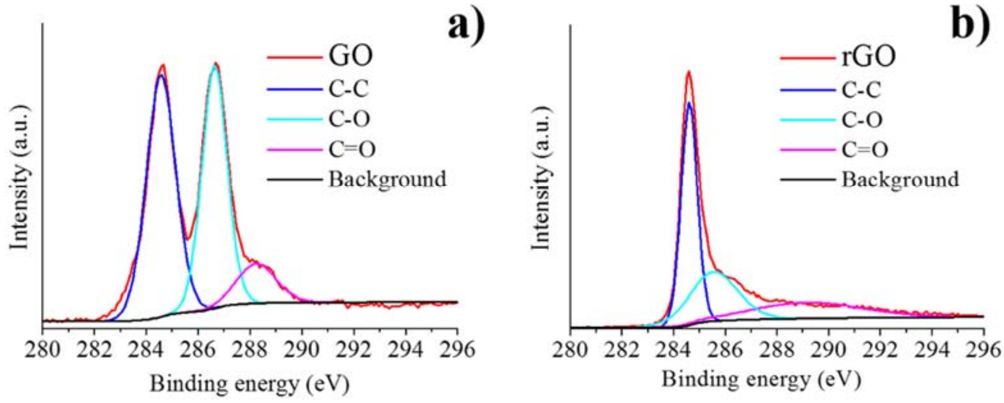

Figure S4. C1s XPS spectra of a) GO and b) rGO.

6. HRTEM of silver nanoparticles on GO obtain by laser ablation.

As a proof of concept, silver nanoparticles (AgNPs) were synthesized by the same experimental approach used in this communication (Figure S5). The results show that short pulsed laser ablation in liquids is a method that can be used for the preparation and immobilization of silver nanoparticles in a single reaction step.

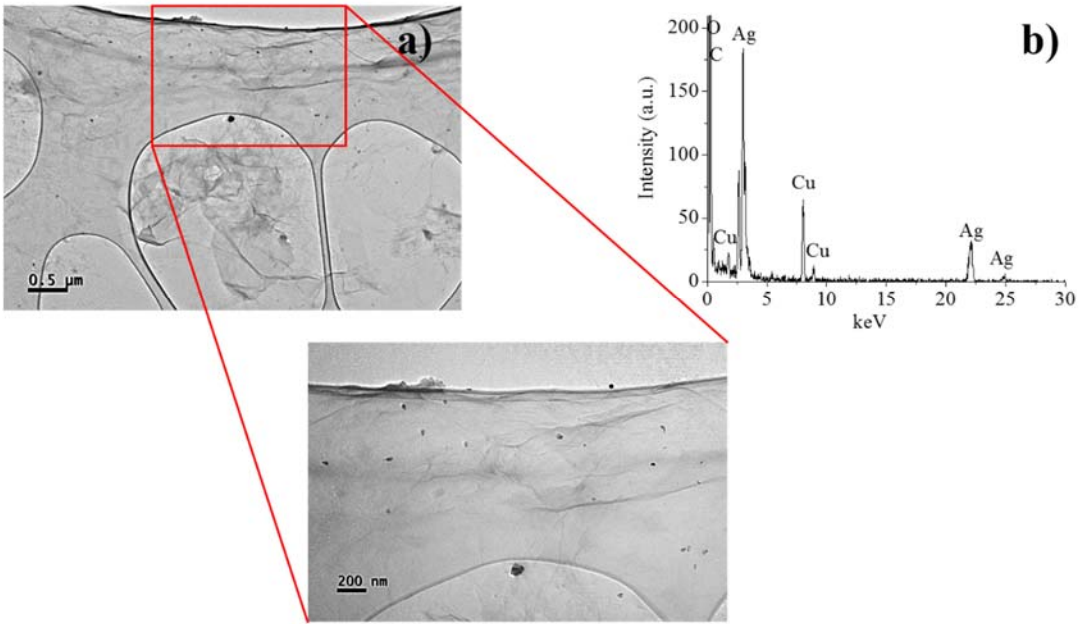

Figure S5. Micrographs of AgNPs anchored in graphene oxide (GO). a) HRTEM micrograph and zoom b) EDX spectrum of the produced AgNPs showing the presence of silver.

7. Supplementary references.

[1] K. Maximova, A. Aristov, M. Sentis & A. V. Kabashin, Size-controllable synthesis of bare gold nanoparticles by femtosecond laser fragmentation in water, Nanotechnology **26**, 065601 (2015).

[2] H. Lim, J. Jung, R. Ruoff & Y. Kim, Structurally driven one-dimensional electron confinement in sub-5-nm graphene nanowrinkles, Nat. Commun. **6**, 8601 (2015).
